# Colectomy and Neoplasia Outcomes of Patients With Ulcerative Colitis Receiving Golimumab: A Post‐Authorisation Safety Study Using the Spanish ENEIDA Registry

**DOI:** 10.1002/pds.70176

**Published:** 2025-07-28

**Authors:** Eugeni Domènech, Joan Fortuny, David Martínez, Anita Tormos, Zhiping Huang, Deanna D. Hill, Cindy Weinstein, Suzan Esslinger, Alexis A. Krumme, Marijo Otero‐Lobato, Daniel Mines, Javier P. Gisbert, the ENEIDA Study Group of GETECCU, Beatriz Sicilia, David Monfort I Miquel, Xavier Calvet Calvo, Miguel Mínguez Pérez, Luis Fernández Salazar, Esther García Planella, Joaquín de Hinojosa de Val, Rufo H. Lorente Poyatos, Luis Bujanda, Mariana Fe Garcia Sepulcre, Ana Gutiérrez Casbas, Eugeni Domènech, Lucía Márquez Mosquera, Maria Dolores Martin Arranz, Javier P. Gisbert, Maria Esteve Comas, Antonio López San Roman, Guillermo Bastida Paz, Jose Manuel Benitez Cantero, Manuel Van Domselaar, Xavier Aldeguer, Fernando Bermejo, Jose Lazaro Perez Calle, Montserrat Rivero Tirado, Santiago Garcia López, Jesús Barrio Andrés, Guillermo Alcaín Martinez, Elena Ricart Goméz, Carla J. Gargallo Puyuelo, Miguel Ángel Montoro Huguet

**Affiliations:** ^1^ Gastroenterology Department Hospital Universitari Germans Trias i Pujol, Autonomous University of Barcelona Barcelona Spain; ^2^ Centro de Investigación Biomédica en Red de Enfermedades Hepáticas y Digestivas (CIBEREHD) Madrid Spain; ^3^ RTI Health Solutions Barcelona Spain; ^4^ Merck & Co., Inc. Rahway New Jersey USA; ^5^ Johnson & Johnson Innovative Medicine Zug Switzerland; ^6^ Johnson & Johnson Titusville New Jersey USA; ^7^ Johnson & Johnson Innovative Medicine Biologics B.V. Leiden the Netherlands; ^8^ RTI Health Solutions Durham North Carolina USA; ^9^ Gastroenterology Unit, Hospital Universitario de La Princesa, Instituto de Investigación Sanitaria Princesa (IIS‐Princesa), Universidad Autónoma de Madrid (UAM) Madrid Spain

**Keywords:** biologics, colectomy, colorectal cancer, epidemiology, inflammatory bowel disease (IBD), ulcerative colitis

## Abstract

**Purpose:**

Golimumab (GLM), an anti‐tumour necrosis factor alpha (anti‐TNFα) agent, is indicated for moderate to severe ulcerative colitis (UC). This post‐authorisation safety study evaluated the risk of colectomy due to intractable disease and advanced colonic neoplasia (high‐grade dysplasia and/or colorectal cancer) under real‐world conditions of GLM use.

**Methods:**

This bidirectional cohort study using Spanish ENEIDA registry data (2013–2022) included adults with UC who initiated GLM, other anti‐TNFα agents, or thiopurines (TPs). Crude risk analyses—and, when feasible, multivariable models—in cohort and nested case‐control designs were performed. For colectomy, we evaluated exposure to GLM only, other anti‐TNFα agents, and both (i.e., overlapping exposure). For ACN, we evaluated exposure to GLM, other anti‐TNFα agents, and TPs.

**Results:**

Sixty‐four colectomy cases and 10 ACN cases were identified among patients exposed to GLM (*N* = 474), other anti‐TNFα agents (*N* = 1737), or TPs (*N* = 1380). Incidence rates per 1000 person‐years and 95% confidence intervals were reported for colectomy (GLM–only [4.4, 1.2–11.2] and other anti‐TNFα agents only [12.4, 9.1–16.5]) and ACN (GLM [1.5, 0.2–5.4], other anti‐TNFα agents [1.3, 0.5–2.8], and TPs [1.0, 0.3–2.6]). In comparisons excluding overlapping exposure, GLM was not associated with an increased risk of colectomy versus other anti‐TNFα agents. GLM was also not associated with an increased risk of ACN versus either comparator. Observed events, especially for ACN, were limited for all exposures.

**Conclusions:**

Findings do not indicate an increased risk of colectomy due to intractable disease or ACN with GLM use versus other therapies for similar disease severity in routine UC care (EUPAS15752).

**Summary**

- When the European Medicines Agency approved golimumab (GLM) (an anti‐tumour necrosis factor alpha [anti‐TNFα] agent) for the treatment of moderate to severe ulcerative colitis (UC) in 2013, long‐term safety outcomes data were limited.
- To fill the gap, this 8.5‐year observational study evaluated the risk of colectomy due to intractable disease and advanced colonic neoplasia (ACN) in patients with UC under real‐world conditions using Spanish ENEIDA registry data.
- Overall, the risk of colectomy due to intractable disease and ACN was not increased in UC patients exposed to GLM versus those exposed to other anti‐TNFα agents.

## 1 Introduction

Ulcerative colitis (UC) is a chronic inflammatory bowel disease (IBD) that affects approximately 5 million people worldwide [1], and up to 15% of patients experience an aggressive disease course [2]. Long‐term, poorly controlled inflammation with UC is associated with colonic dysplasia [3]. High‐grade dysplasia (HGD) increases the risk of progression to colorectal cancer (CRC) [4]. Compared with the general population, patients with UC have an increased risk of developing both dysplasia and CRC [3, 4].

UC treatment depends on disease extent and severity [5]. For patients with moderate to severe UC, corticosteroids are used to achieve initial disease control, after which thiopurines (TPs) may be prescribed [5]. Those who do not respond to or are intolerant of corticosteroids or TPs may be prescribed an anti‐tumour necrosis factor α (anti‐TNFα) agent as the next line of treatment [6]. If medical therapy ultimately fails to control disease activity, or in some cases of HGD and CRC, colectomy is often the preferred therapeutic approach [7, 8]. Although control of inflammation can decrease the risk of dysplasia [9, 10], immunosuppression can also affect host defences against malignancies [11]. Thus, anti‐TNFα treatment carries a theoretical risk of dysplasia, CRC, and hepatosplenic T‐cell lymphoma (HSTCL) [12, 13].

Golimumab (GLM), an anti‐TNFα agent, was approved to treat moderately to severely active UC in 2013 [6]. This post‐authorisation safety study (PASS) (EUPAS15752, European Medicines Agency [EMA] study ID 50619) [14] was conducted at the request of the EMA) [15] to further evaluate safety outcomes relevant to UC patients under real‐world conditions of GLM use. In alignment with the Pharmacovigilance Risk Assessment Committee's request on behalf of the EMA, data from ENEIDA, a large, prospectively maintained registry of patients with IBD in Spain [16], were used to primarily evaluate the risk of colectomy due to intractable (i.e., difficult‐to‐control) disease and the risk of advanced colonic neoplasia (ACN) (a composite of HGD and/or CRC) among patients with UC who were exposed to GLM versus those who were exposed to alternative therapies for similar disease severity.

## 2 Methods

### 2.1 Study Design and Setting

This long‐term, non‐interventional, observational PASS used a new‐user, bidirectional cohort study design with secondary nested case‐control (NCC) analyses. Cohort analyses used UC‐patient data prospectively collected from 30 ENEIDA sites, and the NCC analyses included data abstracted retrospectively from medical charts at 28 ENEIDA sites. Participating ENEIDA investigators are listed in the Appendix A. The study started on 19 September 2013 (the date GLM received European marketing authorisation for UC), inclusion of new patients ended on 31 December 2021, and assessment of incident outcomes ended on 30 March 2022.

The study protocol (MK‐8259‐042) was approved by the EMA's [14] Pharmacovigilance Risk Assessment Committee, the Spanish Working Group on Crohn's Disease and UC (the scientific society maintaining ENEIDA) [16], the study's reference ethics committee (Hospital Universitario de La Princesa, Madrid), and the Spanish Agency for Medicines and Medical Devices. Patients enrolled in ENEIDA provided written informed consent prior to inclusion of their health information in the registry—including deidentified data collected through the chart abstraction questionnaire—and its use for research purposes. Institutional review boards at all participating sites confirmed the study's chart review was covered by ENEIDAs informed consent process. The STROBE guidelines [17] for reporting observational studies were followed (Table S1).

### 2.2 Participants

This study used all available data from participating ENEIDA sites for patients who met the eligibility criteria in Table S2. Briefly, participants were aged ≥ 18 years, were diagnosed with UC, had no reported history of any study outcomes before cohort entry, and had initiated a cohort‐defining study therapy (GLM, other anti‐TNFα agent, or TP). Cohort entry, including due to switching, corresponded to the date of first recorded use (i.e., baseline, based on the start of the prescribed treatment course) of one of the cohort‐defining therapies. Follow‐up ended on the date of the earliest censoring event encountered (e.g., occurrence of a study outcome, end of study period, loss of follow‐up, and initiation of treatments that became available after 2013; criteria listed in Table S3). For the NCC analyses, potential control patients (i.e., participants without a study outcome) were identified from risk sets assembled using incidence density sampling [18]. Up to 2 control patients who had been exposed to a relevant study therapy within the applicable risk window before the reference date (i.e., outcome date or equivalent) were matched to each case (i.e., patients with a study outcome) on the basis of UC duration and length of follow‐up time at the reference date.

### 2.3 Outcomes and Exposures

Primary outcomes included the incidence of colectomy due to intractable disease (hereafter ‘colectomy’) and the incidence of ACN. Incidence of CRC was a secondary outcome, and incidence of HSTCL was an exploratory outcome. Outcome definitions are in Table S4. GLM, the main exposure of interest, was compared with exposure to other anti‐TNFα agents (i.e., infliximab and adalimumab, including biosimilars) and/or TP analogues (i.e., azathioprine and mercaptopurine). Exposure status was considered \time‐varying. Because patients could qualify as new users (i.e., those without prior prescription records of the same drug or its biosimilar) of each study therapy during the study, a patient could contribute follow‐up time to > 1 exposure category.

**FIGURE 1:**
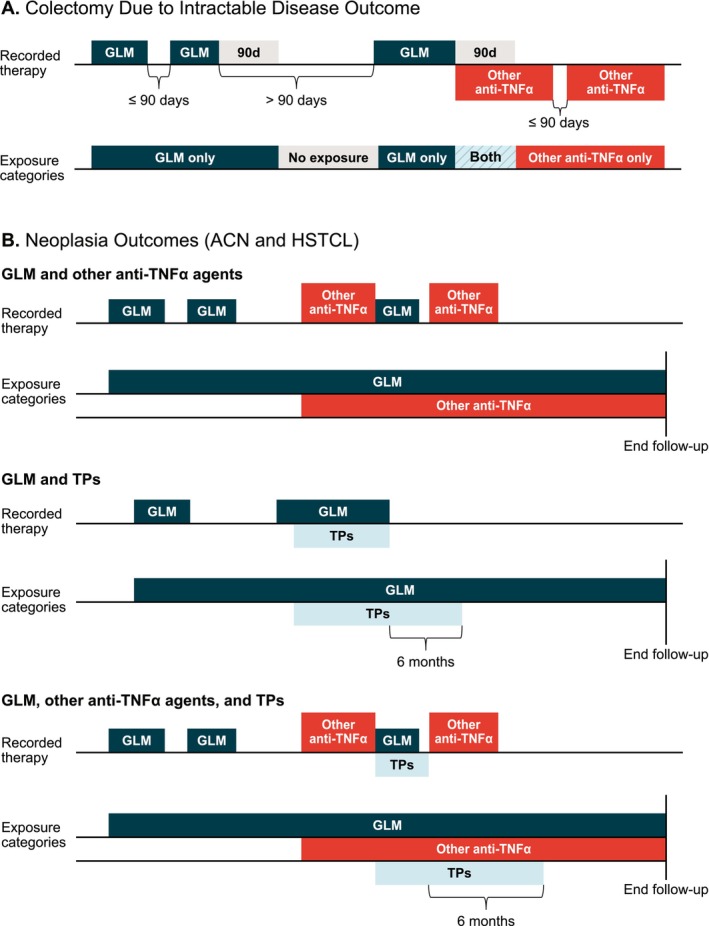
Examples of exposure category definitions. ACN, advanced colonic neoplasia; d, number of days; GLM, golimumab; HSTCL, hepatosplenic T‐cell lymphoma; TNFα, tumour necrosis factor α; TPs, thiopurines. For the outcome of colectomy due to intractable disease, time at risk started 1 day after cohort entry and continued to 90 days after the last treatment or end of follow‐up, whichever occurred first. Periods of overlapping exposure, thus, were possible if one therapy was started shortly after discontinuing the other one. Therefore, we used 3 categories in the analyses of the risk of colectomy: GLM only, other anti‐TNFα only, and both. Given that the effect of the study therapies could theoretically last long after the end of exposure, the analysis of neoplasia outcomes used a different risk window (as noted in the lower half of the figure). For the cohorts exposed to GLM or other anti‐TNFα agents, the risk window extended to the end of follow‐up, whereas the risk window for the cohort exposed to TPs extended 6 months after treatment discontinuation. Given the definition of these risk windows, a patient who switched therapies during the study could contribute person‐time to > 1 exposure category simultaneously.

Figure 1 depicts exposure categories. As the timing of potential biologic effects of study therapies may differ among outcomes, outcome‐specific risk windows were used. The colectomy analysis used 3 mutually exclusive exposure categories: GLM only, other anti‐TNFα agents only, and overlapping exposure to both GLM and other anti‐TNFα therapies. Exposure categories for the neoplasia outcomes (ACN, CRC only and HSTCL) included GLM, other anti‐TNFα agents, and TPs.

### 2.4 Data Sources and Measurement

This study involved secondary analysis of data from the ENEIDA database [16], which documents the care of patients with IBD in a routine clinical practice setting. ENEIDA sites are expected to collect a set of mandatory variables for each registered patient. Sites that enrolled ≥ 75% of their patients with IBD (a self‐reported variable) into the registry and entered ≥ 75% of the mandatory variables on the ENEIDA electronic case report forms were determined to have research‐quality data and were selected to provide data for this study. Of the 191 chart abstractions requested for the NCC analyses, 178 completed questionnaires were obtained. An exploratory analysis substantiated the validity of using the abstracted information for the stated study objectives, supporting this study's methodology (Table S5).

### 2.5 Statistical Methods

Data were analysed using SAS version 9.4 or higher (SAS Institute; 2013). Patients without a recorded diagnosis for the outcomes evaluated were considered to not have the condition, and missing data were not imputed. No a priori hypotheses were evaluated.

#### 2.5.1 Cohort Analyses

Cohorts were characterised using standard descriptive statistics. Incidence rates (IRs) were calculated as the number of outcomes divided by the person‐years (PYs) at risk. Univariable Poisson and Cox regression models were generated for the colectomy outcome (methods are expanded in Table S4). Cox models were also generated for ACN and CRC, but not for HSTCL, due to the absence of events. To guide the selection of potential confounding variables, crude IR ratio (IRR) associations were compared with Mantel‐Haenszel adjusted IRRs for each candidate variable (screening and selection methods are in Table S6, and related data are in Table S7). Candidate covariates were evaluated for colectomy and ACN outcomes but not for CRC or HSTCL, for which sparse (*n*   <  10) outcomes did not support variable adjustment [19]. Planned subgroup (described below) and sensitivity (using an alternative risk window of exposure through end of follow‐up [i.e., ‘ever exposed, always at risk’] [20, 21, 22]) analyses were conducted for the colectomy outcome only. In accordance with the prespecified plan, these analyses were not conducted for sparse outcomes. To explore possible competing risks [23] between the colectomy and ACN outcomes, a multivariable Cox regression model was generated using a composite endpoint—time to the earliest of colectomy, ACN, or death—in an ‘ever‐exposed, always‐at‐risk’ [20, 21, 22] approach.

#### 2.5.2 NCC Analyses

Crude odds ratios (ORs) were estimated using a univariable conditional logistic regression model. After the covariates for adjustment were determined (screening and selection methods are in Table S6, and related data are in Table S8), multivariable logistic regression—conditional on matching—to estimate adjusted ORs was used. Because CRC events were sparse, NCC analyses were conducted only for the colectomy (crude and adjusted ORs) and ACN (crude OR) outcomes.

## 3 Results

### 3.1 Participants

Across the 30 participating ENEIDA sites, there were 19 738 patients with UC. After applying study eligibility criteria (Table S9), the study included 474 patients who initiated GLM, 1737 who initiated other anti‐TNFα agents, and 1380 who initiated TPs. Table 1 summarises patient demographic and clinical characteristics at cohort entry. Age at entry was similar across the 3 cohorts, but the GLM cohort tended to have the longest UC duration and was more likely than the other anti‐TNFα agents cohort to have previously used ≥ 1 anti‐TNFα therapy. Mean follow‐up time for each outcome was similar across cohorts (colectomy range, 23–26 months; ACN range, 32–34 months; HSTCL range, 33–35 months) (Table S10) and, for all analyses, the most common reasons for censoring were the end of study period, loss of follow‐up, and use of novel biologic agents (Table S11). The NCC analyses included 41 cases with 70 matched controls for the colectomy outcome and 9 cases with 11 controls for ACN (characteristics listed in Table S12).

**TABLE 1:** Patient characteristics at cohort entry.

Characteristic	Study cohort		
	GLM (*N* = 474)	Other anti‐TNFα agents (*N* = 1737)	TPs (*N* = 1380)
Age in years			
*n*	474	1737	1380
Mean (SD)	45.1 (14.1)	44.2 (153)	43.1 (14.8)
Median (Q1, Q3)	44.0 (36.0, 55.0)	44.0 (33.0, 55.0)	42.0 (31.0, 54.0)
Min, max	18.0, 87.0	18.0, 98.0	18.0, 92.0
Age group, *n* (%)			
18 to < 35 years	114 (24.1)	510 (29.4)	422 (30.6)
35 to < 65 years	319 (67.3)	1047 (60.3)	839 (60.8)
≥ 65 years	41 (8.6)	180 (10.4)	119 (8.6)
Sex, *n* (%)			
Male	236 (49.8)	910 (52.4)	740 (53.6)
Female	238 (50.2)	827 (47.6)	640 (46.4)
Calendar year of cohort entry, *n* (% over total new use of the study therapies [column%]/% over total yearly new use of study therapies [row%])			
2013	2 (0.4/1.6)	64 (3.7/51.2)	59 (4.3/47.2)
2014	77 (16.2/15.0)	203 (11.7/39.6)	233 (16.9/45.4)
2015	67 (14.1/12.1)	238 (13.7/43.1)	247 (17.9/44.7)
2016	71 (15.0/13.7)	232 (13.4/44.9)	214 (15.5/41.4)
2017	75 (15.8/15.8)	232 (13.4/48.7)	169 (12.2/35.5)
2018	63 (13.3/16.0)	196 (11.3/49.7)	135 (9.8/34.3)
2019	58 (12.2/14.8)	202 (11.6/51.4)	133 (9.6/33.8)
2020	35 (7.4/10.8)	188 (10.8/58.0)	101 (7.3/31.2)
2021	26 (5.5/8.8)	182 (10.5/61.3)	89 (6.4/30.0)
UC duration in years^a^			
*n*	474	1737	1380
Mean (SD)	8.9 (8.1)	7.2 (7.8)	5.4 (6.7)
Median (Q1, Q3)	6.6 (2.2, 13.1)	4.1 (1.2, 11.0)	2.5 (0.7, 8.0)
Min, max	0.0, 41.0	0.0, 42.6	0.0, 39.4
Maximum extent of disease, *n* (%)^b^			
Extensive	232 (48.9)	899 (51.8)	672 (48.7)
Left side only	190 (40.1)	633 (36.4)	549 (39.8)
Proctitis	29 (6.1)	109 (6.3)	101 (7.3)
Not recorded	23 (4.9)	96 (5.5)	58 (4.2)
Prior treatment with steroids, *n* (%)^c^			
No	197 (41.6)	635 (36.6)	527 (38.2)
Yes	277 (58.4)	1102 (63.4)	853 (61.8)
Prior treatment with cyclosporine, *n* (%)^c^			
No	449 (94.7)	1628 (93.7)	1317 (95.4)
Yes	25 (5.3)	109 (6.3)	63 (4.6)
Hospitalised for UC, *n* (%)^c^			
No	380 (80.2)	1358 (78.2)	1178 (85.4)
Yes	94 (19.8)	379 (21.8)	202 (14.6)
Prior diagnosis with PSC, *n* (%)^d^			
No	177 (37.3)	469 (27.0)	261 (18.9)
Yes	5 (1.1)	19 (1.1)	20 (1.4)
Unknown/missing	292 (61.6)	1249 (71.9)	1099 (79.6)
Prior screening colonoscopy, *n* (%)^c^			
No	363 (76.6)	1442 (83.0)	1194 (86.5)
Yes	111 (23.4)	295 (17.0)	186 (13.5)
Number of previous other anti‐TNFα agents, *n* (%)^c^			
0	307 (64.8)	1503 (86.5)	1380 (100)
1	113 (23.8)	227 (13.1)	0 (0)
2	54 (11.4)	7 (0.4)	0 (0)
Recent switcher after use of another antiTNFα agent, *n* (%)^e^			
After short‐term use (≤ 3 months)	109 (23.0)	165 (9.5)	0 (0)
After long‐term use (> 3 months)	50 (10.5)	49 (2.8)	0 (0)
Did not switch	315 (66.5)	1523 (87.7)	0 (0)

*Note:* Study cohorts were not mutually exclusive. Patients could qualify for > 1 cohort if they met all applicable criteria. Cohort entry, including due to switching, corresponded to the date of first recorded use (i.e., baseline, based on the start of the prescribed treatment course) of one of the cohort‐defining therapies.

Abbreviations: GLM, golimumab; PSC, primary sclerosing cholangitis; Q1, first quartile; Q3, third quartile; SD, standard deviation; TNFα, tumour necrosis factor α; TP, thiopurine; UC, ulcerative colitis.

^a^ UC duration was the time between the date of UC diagnosis and cohort entry date.

^b^ Maximum extent of disease reflects the value for this patient ascertained on 30 March 2022, when data extraction occurred. This value may not reflect the actual maximal disease extent at the time of cohort entry. In ENEIDA data, there is only 1 value of this variable per patient; this variable is not date stamped and is subject to continual updating to reflect the maximum extent of disease reached.

^c^ During the period from UC diagnosis to the cohort entry date.

^d^ Ever before the cohort entry date.

^e^ Recent switcher refers to a patient who started a new anti‐TNFα agent within 90 days of discontinuing another anti‐TNFα agent.

For the colectomy outcome, demographic and clinical characteristics of the 185 patients with overlapping exposure to both GLM and other anti‐TNFα agents are noted in Table S13. These patients were all, by definition, recent switchers (i.e., they entered the overlapping use exposure category because they switched between cohorts during the 90‐day period since the last recorded use of the discontinued drug). Patients in the overlapping exposure category had a higher prevalence of prior hospitalizations for UC (24.9%) and prior use of ≥ 2 anti‐TNFα therapies (17.9%) than those who were in the GLM‐only cohort (19.8% and 11.4%, respectively) or were in the other anti‐TNFα agents‐only cohort (21.8% and 0.4%, respectively). Other demographic and clinical characteristics were similar at cohort entry.

### 3.2 Colectomy due to Intractable Disease

There were 64 unique colectomy events across all exposure categories (Table 2). The crude colectomy IR was lowest during exposure to GLM only and highest during overlapping exposure to both GLM and other anti‐TNFα therapies (Figure S1 shows cumulative incidences). In crude and adjusted cohort analyses, relative risk estimates comparing GLM‐only use with other anti‐TNFα agents‐only use were not elevated (Table 3). However, based on 3 events, the risk of colectomy was greater in the overlapping exposure group than in the GLM‐only and other anti‐TNFα agents‐only groups. Results from the unadjusted subgroup analyses were like those of the main analyses. Specifically, GLM‐only use was not associated with an increased risk of colectomy in analyses that were stratified on prior use of anti‐TNFα agents or first therapy in the comparator cohort (either infliximab or adalimumab). In analyses among those with concomitant TP use at baseline, the risk was similar with GLM only versus other anti‐TNFα agents only (hazard ratio [HR], 1.06; 95% confidence interval [CI], 0.30‐3.67), but GLM only was not associated with an increased risk versus the overlapping exposure group. In analyses among those without concomitant TP use at baseline, GLM‐only use was also not associated with an increased risk of colectomy (Table S14). Additionally, in sensitivity analyses using an alternative follow‐up consisting of an ‘ever‐exposed, always‐at‐risk’ approach, models showed directionally consistent colectomy risk estimates for GLM versus other anti‐TNFα therapies. Similarly, in crude and adjusted NCC analyses, GLM‐only use (*n* = 3 events) had a lower risk of colectomy versus other anti‐TNFα agents−only use (*n* = 35 events), though overlapping exposure to both (*n* = 3 events) showed an increased risk of colectomy in crude and adjusted analyses compared with GLM‐only or other anti‐TNFα agents‐only use.

**TABLE 2:** Crude IRs for colectomy due to intractable disease by mutually exclusive exposure category.

Statistic	Exposure category			
	GLM only	Other anti‐TNFα agents only	GLM + other anti‐TNFα agents	No anti‐TNFα agent exposure^a^
*n*	4	47	3	10
PY	912.3	3791.9	38.2	770.2
IR per 1000 PYs	4.4	12.4	78.6	13.0
95% CI	1.2–11.2	9.1–16.5	16.2–229.7	6.3–23.9

*Note:* PYs were calculated based on risk windows. Because patients may have changed therapy during the study, patients may have contributed to > 1 exposure category during follow‐up.

Abbreviations: CI, confidence interval; GLM, golimumab; IR, incidence rate; PY, person‐year; TNFα, tumour necrosis factor α.

^a^ Refers to person‐time when there was no exposure to GLM or other anti‐TNFα agents between episodes of GLM or other anti‐TNFα agent use.

**TABLE 3:** Summary of comparative analyses for colectomy due to intractable disease, ACN, and CRC.

Outcome	Exposure comparison	Number of events	Cohort analyses				NCC analyses	
			Poisson model		Cox model		Conditional logistic model	
			Crude IRR (95% CI)	Adjusted IRR (95% CI)	Crude HR (95% CI)	Adjusted HR (95% CI)	Crude OR (95% CI)	Adjusted OR (95% CI)
Colectomy due to intractable disease^a^	GLM only vs. other anti‐TNFα agents only	Cohort: 4 vs. 47 NCC: 3 vs. 35	0.35 (0.13–0.98)	0.40 (0.14–1.13)	0.37 (0.13–1.02)	0.41 (0.15–1.15)	0.17 (0.04–0.78)	0.16 (0.02–1.08)
	GLM only vs. (GLM + other anti‐TNFα agents)	Cohort: 4 vs. 3 NCC: 3 vs. 3	0.06 (0.01–0.25)	0.06 (0.01–0.26)	0.07 (0.02–0.31)	0.08 (0.02–0.38)	0.04 (0.00‐NE)	0.02 (0.00–0.50)
	(GLM + other anti‐TNFα agents) vs. other anti‐TNFα agents–only	Cohort: 3 vs. 47 NCC: 3 vs. 35	6.34 (1.97–20.37)	6.78 (2.08–22.13)	5.33 (1.64–17.34)	4.95 (1.52–16.08)	4.03 (0.40‐NE)	8.69 (0.60–124.90)
ACN^b^	GLM vs. other anti‐TNFα agents	Cohort: 2 vs. 6 NCC: 1 vs. 5	NP	NP	1.16 (0.23–5.73)	1.09 (0.22–5.44)	0.47 (0.04–5.68)	NP
	GLM vs. TP	Cohort: 2 vs. 4 NCC: 1 vs. 3	NP	NP	1.46 (0.27–8.00)	1.08 (0.19–6.13)	0.42 (0.01–13.27)	NP
CRC^c^	GLM vs. other anti‐TNFα agents	Cohort: 2 vs. 2 NCC: NP	NP	NP	3.42 (0.48–24.26)	NP	NP	NP
	GLM vs. TP	Cohort: 2 vs. 3 NCC: NP	NP	NP	1.92 (0.32–11.50)	NP	NP	NP

Abbreviations: ACN, advanced colonic neoplasia; CI, confidence interval; CRC, colorectal cancer; GLM, golimumab; HR, hazard ratio; IRR, incidence rate ratio; NCC, nested case–control; NE, not estimable; NP, not performed; OR, odds ratio, TNFα, tumour necrosis factor α; TP, thiopurine; UC, ulcerative colitis.

^a^ Poisson and Cox models were adjusted for age group (dichotomised ≥ 35 years and < 35 years), UC duration, prior treatment with cyclosporine, and sex. The conditional logistic model was adjusted for age (dichotomised ≥ 35 years and < 35 years), any hospitalisation in the year before the last episode of a study therapy commenced, cyclosporine use in the year before the last episode of a study therapy commenced, and sex.

^b^ Cox models were adjusted for age group (3 categories), UC duration, and prior treatment with cyclosporine. Exposure categories were not mutually exclusive.

^c^ Statistics are by study exposure and use the applicable risk window of follow‐up time specific to the outcome. Results were derived from a single univariable Cox regression model. Only exposure categories present in the data were included in regression models. Exposure categories were not mutually exclusive.

### 3.3 Neoplasia Outcomes

There were 10 unique ACN and 6 unique CRC events. The crude IR of ACN ranged from 1.0 to 1.5 per 1000 PYs, and the IR of CRC ranged from 0.4 to 1.5 per 1000 PYs (Table 4; Figure S1 shows cumulative incidences). No HSTCL cases were identified. In crude and adjusted cohort analyses and in NCC analyses (based on 1 GLM‐exposed case), GLM use was not associated with a higher risk of ACN versus use of other anti‐TNFα or TP therapies. In crude comparative analyses of CRC, GLM use was associated with a higher HR versus use of other anti‐TNFα and TP therapies; however, the CIs were wide and included unity, and comparisons were based on sparse events (GLM, 2; other anti‐TNFα agents, 2; TPs, 3). In the competing risk analysis, GLM use, versus use of other anti‐TNFα therapies, was not associated with an increased risk of the composite outcome (i.e., colectomy due to intractable disease, ACN, and/or death). The exposure to GLM versus exposure to TPs was associated with an increased risk in the composite outcome (HR, 1.78; 95% CI, 0.93‐3.41) (Table S15).

**TABLE 4:** Crude IRs for neoplasia outcomes, by exposure category.

Outcome	Exposure category		
	GLM	Other anti‐TNFα agents	TP
ACN			
*n* ^a^	2	6	4
PYs	1347.8	4637.2	3871.8
IR per 1000 PYs	1.5	1.3	1.0
95% CI	0.2–5.4	0.5–2.8	0.3–2.6
CRC			
*n* ^b^	2	2	3
PYs	1347.8	4637.2	3871.8
IR per 1000 PYs	1.5	0.4	0.8
95% CI	0.2–5.4	0.1–1.6	0.2–2.3
HSTCL			
*n*	0	0	0
PYs	1393.2	4798.9	3892.0
IR per 1000 PYs	0.0	0.0	0.0
95% CI	0.0–2.6	0.0–0.8	0.0–0.9

*Note:* PYs at risk were specific to each outcome and based on the applicable risk window for each outcome. Patients could have been exposed to > 1 study therapy during the course of follow‐up; because risk windows could overlap, a single outcome could be attributed to > 1 exposure category.

Abbreviations: ACN, advanced colorectal neoplasia; CI, confidence interval; CRC, colorectal cancer; GLM, golimumab; HSTCL, hepatosplenic T‐cell lymphoma; IR, incidence rate; PY, person‐year; TNFα, tumour necrosis factor α; TP, thiopurine.

^a^ There were 10 unique occurrences of ACN across all exposure categories. For 2 affected patients, the ACN occurred during risk windows attributable to > 1 exposure category.

^b^ There were 6 unique occurrences of CRC across all exposure categories. In 1 patient, CRC occurred during risk windows attributable to > 1 exposure category.

## 4 Discussion

This PASS evaluated the risk of safety outcomes among new users of GLM for UC based on ENEIDA registry data. During the study period, 19 738 patients with UC from geographically diverse research‐quality centres were available in the registry. The number of contributing hospitals, including several large tertiary‐care university hospitals and a variety of large and medium‐sized hospitals and clinics, indicates that the data herein are representative of the clinical practice among patients with moderate to severe UC in Spain [16]. ENEIDA provided quality data on both the exposures and outcomes of interest and had a very high response rate (93%) to the questionnaires for medical chart abstraction.

Overall, there were 64 unique colectomy events and 6 unique CRC events among 10 unique ACN events identified during the 8.5‐year study period. No HSTCL events were identified, which was expected given its rarity [24]. At baseline, patients in the GLM cohort and other anti‐TNFα agents cohort appeared to have a history of more severe UC (based on proxies of prior reported disease severity, longer UC duration, and more hospitalisations for UC) than those in the TP cohort who were anti‐TNFα agent–naive. Additionally, new users of GLM tended to have switched from another anti‐TNFα agent more often than new users of other anti‐TNFα agents, suggesting that GLM may have been used preferentially after a course of another anti‐TNFα therapy.

Colectomy IRs per 1000 PYs appeared lower with GLM‐only use (4.4) versus other anti‐TNFα agents‐only use (12.4), although the CIs overlapped. Notably, the colectomy IR with GLM‐only use was consistent with those reported from multiple large, contemporaneous, observational studies of patients with UC (per 1000 PYs): 4.2 in the United States (2016) [25]; 4.4 in Scotland (2018) [26]; and 1.9–4.2 in South Korea (2010–2016) [27]. Conversely, patients exposed only to other anti‐TNFα agents had higher colectomy IRs than those reported in the literature [25, 26, 27]. This may be due to the preferential use of infliximab in patients with severe UC flares, who may have increased colectomy risk. The subgroup analysis result supports this hypothesis; specifically, when the first therapy in the other anti‐TNFα cohort was infliximab, there was a stronger inverse association of GLM exposure with colectomy than when the first therapy in the other anti‐TNFα cohort was adalimumab.

Overall, GLM use was not associated with an increased risk of colectomy, and risk estimates are consistent in subgroup and sensitivity analyses. However, overlapping exposure to both GLM and other anti‐TNFα agents was associated with a higher risk of colectomy than exposure to GLM only or other anti‐TNFα agents only, although results were based on only 3 events. Overlapping exposure to both GLM and other anti‐TNFα agents occurred during periods when patients switched from one therapy to another. Thus, it is possible that the elevated colectomy risk during this period can be explained by the clinical factors that prompted the switch rather than therapy effects, a notion that we cannot formally evaluate without any direct measurements of disease activity. Adjustment for measured covariates, some of which were proxies of disease activity, only minimally attenuated the risk of colectomy during overlapping exposure. Potential confounding by use of newer biologics that were introduced during the study period were addressed by design (exclusion) rather than by adjustment. Still, it is important to note that unadjusted analyses restricted to those who were naive to anti‐TNFα agents suggest that the risk of colectomy was similar among users of GLM only and other anti‐TNFα agents only, reinforcing the notion that GLM does not increase the risk of colectomy.

In our study (based on 10 ACN events), compared with the use of other anti‐TNFα or TP therapies, GLM use was not associated with an increased risk of ACN in either the adjusted Cox model or the NCC analyses. To our knowledge, only 1 cohort study has used a similar ACN case definition in an unselected UC population (also from ENEIDA); however, it did not report an IR to compare with our results ranging from 1 to 1.5 per 1000 PYs, but rather included a cumulative incidence based on variable amounts of follow‐up time for each participant [9]. Lastly, in evaluating the potential for competing risks, GLM use was not associated with a significantly increased risk of the composite outcome (i.e., colectomy due to intractable disease, ACN and/or death)—versus the use of other anti‐TNFα agents—and did not alter the interpretation of the study.

Our IR estimates of CRC per 1000 PYs (GLM, 1.5; other anti‐TNFα agents, 0.4; TPs, 0.8) are compatible with those from recent population‐based studies of CRC in UC (IR = 1.24 [28] and IR = 1.29 [29] per 1000 PYs). Although the point estimates in our study were elevated for GLM use versus use of anti‐TNFα or TP therapies, they were imprecise and based upon comparisons of sparse events. In addition to random variation, these associations might also be explained by confounding; adjusted analyses could not be conducted due to limited events.

Our study had several potential limitations. Although the study design and finalised protocol reflect the UC treatment landscape in 2015, we believe that our study has robust internal validity. This assertion is supported by the consistency of our results across analytical methods. ENEIDA data on the main study outcomes was reasonably complete, and chart review captured information on potential confounding variables in the NCC analyses. Of note, ACN is commonly asymptomatic and is often identified only during a screening colonoscopy. Since ENEIDA data reflect routine clinical practice, and patients were not required to participate in CRC screening programmes, the true number of ACN events may have been underestimated. It is also possible that better control of inflammation might decrease ACN risk [30, 31], though this cannot be concluded here because we did not include patients without immunosuppressive therapy use. It should be acknowledged that survival prior to GLM could have theoretically led to a depletion of susceptibles, meaning outcomes may not reflect the true risk for the entire population. However, this is unlikely to have impacted our results, as cohort analyses were adjusted for UC severity markers (such as the duration of UC) at the index date, and the change in the various relative risk estimates after adjustment did not indicate a strong confounding effect of the variables used for adjustment. Also, the distribution of UC severity markers was similar between groups, and the proportion of patients censored due to switching to a novel treatment (a likely marker of uncontrolled disease) was numerically higher in the GLM cohort versus the anti‐TNFα agents cohort. Lastly, both GLM and anti‐TNFα agents are indicated for use in the same line of therapy for UC.

Because it was recognised during study design that disease activity could not be measured directly, several proxies for prior reported disease severity were used (e.g., disease extent, disease duration, UC hospitalisation history, UC treatment history). However, the sparsity of outcomes constrained our ability to adjust for many potential confounders simultaneously, raising the question of residual confounding bias. Adjusting for a limited number of variables most strongly associated with outcomes had minimal effect on the point estimates, suggesting the absence of important confounding of our results by measured factors. As is true in all observational research, residual confounding by unmeasured factors cannot be definitively excluded. The sparsity of outcomes may have limited detection of small risk increases and restricted the ability to perform additional analyses (e.g., stratification by UC duration). Also, comparisons with other newer biologics (e.g., vedolizumab) were not assessed, as they were not marketed at the study start. Although results from this study may not be generalisable to studies using alternative outcome definitions, the IR estimates are in line with published literature [25, 26, 27, 28, 29], including those of a recently published manuscript on the risk of colectomy due to intractable disease among GLM users in Denmark and Sweden [32], and support the outcomes data obtained from ENEIDA.

## 5 Conclusion

This PASS, based upon a prospectively followed population of patients with UC under routine clinical care in Spain, does not indicate that GLM exposure is associated with a higher risk of colectomy due to intractable disease compared with exposure to other anti‐TNFα therapies. Results were consistent across different analytical methods as well as across subgroup and sensitivity analyses. ACN results, though based on limited events, do not suggest an increased risk among GLM users versus users of other anti‐TNFα or TP therapies. No conclusion could be made about CRC or HSTCL risk due to the limited number or absence of events, respectively.

### 5.1 Plain Language Summary

Patients with ulcerative colitis often receive therapies that reduce inflammation, such as anti‐tumour necrosis factor alpha (anti‐TNFα) therapies. Golimumab is an anti‐TNFα therapy that was approved to treat patients with moderate to severe ulcerative colitis in 2013. In this 8.5‐year study of adult patients with ulcerative colitis in Spain, we gathered information on whether patients treated with golimumab were more likely than those treated with other anti‐TNFα therapies to require colon‐removal surgery (‘colectomy’) due to uncontrollable disease or to develop a composite of colon pre‐cancerous lesions and/or colon cancer (‘advanced colonic neoplasia’). Using disease registry data, we measured how often patients exposed to golimumab underwent colectomy and how often they developed advanced colonic neoplasia. We then compared the occurrence of these events among similar patients with ulcerative colitis who were exposed to other anti‐TNFα therapies. Overall, we found 64 cases of colectomy and 10 cases of advanced colonic neoplasia among all 3591 patients included in this study. The results did not indicate that patients exposed to golimumab had an increased risk of colectomy or of advanced colonic neoplasia compared with patients exposed to other anti‐TNF therapies.

## Author Contributions

Eugeni Domènech, Joan Fortuny, Zhiping Huang, Marijo Otero‐Lobato, Daniel Mines, and Javier P. Gisbert contributed to the conception and design of the study; Eugeni Domènech, David Martínez, Anita Tormos, and Javier P. Gisbert contributed to the acquisition of data; Joan Fortuny, David Martínez, Daniel Mines, and Javier P. Gisbert contributed to the analysis of the data; and Eugeni Domènech, Joan Fortuny, David Martínez, Zhiping Huang, Deanna D. Hill, Cindy Weinstein, Suzan Esslinger, Alexis A. Krumme, Marijo Otero‐Lobato, Daniel Mines, and Javier P. Gisbert contributed to the interpretation of data. All authors drafted the article and/or revised it critically for important intellectual content. Lastly, all authors had full access to the data in the study, approved the final version of the manuscript to be submitted for publication, and agreed to be accountable for all aspects of the work.

## Ethics Statement

The study protocol was reviewed and approved by the European Medicines Agency on 22 October 2015, GETECCU (the scientific society maintaining the ENEIDA registry) on 19 September 2016, the study's reference ethics committee (Hospital Universitario de La Princesa, Madrid) on 16 May 2016, and the Spanish Agency for Medicines and Medical Devices on 13 September 2016. This study also included a nested case‐control analysis involving medical chart abstraction that required specific additional institutional review board (IRB) reviews. At all participating sites, IRBs confirmed that the ENEIDA registry informed consent also covered the chart review portion of the study because all requested variables were within the scope of information collected by the registry. Identifiable patient‐level data remained entirely within the ENEIDA environment, and RTI Health Solutions personnel had access to only the deidentified ENEIDA data necessary to conduct the study and to the deidentified data abstracted from medical charts. The study protocol conformed to the ethical guidelines of the 1975 Declaration of Helsinki.

## Consent

To be enrolled in the ENEIDA registry, patients needed to sign an informed consent form to allow inclusion of their health information in the registry and its use for research purposes. No study‐specific informed consent form was required for the use of the routine ENEIDA data.

## Conflicts of Interest

Eugeni Domènech has served as a speaker, has received research or education funding, or has received advisory fees from AbbVie, Adacyte Therapeutics, Biogen, Celltrion, Ferring, Galapagos, Gilead, GoodGut, IMIDomics, Janssen, Kern Pharma, Eli Lilly, MSD, Pfizer, Roche, Samsung, Takeda, and Tillotts. Joan Fortuny, David Martínez, and Anita Tormos are full‐time employees of RTI Health Solutions, an independent nonprofit research organisation, which was retained by Merck Sharp & Dohme LLC, a subsidiary of Merck & Co., Inc. Rahway, New Jersey, USA, to conduct the research that is the subject of this manuscript. Their compensation is unconnected to the studies on which they work. Zhiping Huang, Deanna D. Hill, and Cindy Weinstein are employees of Merck Sharp & Dohme LLC, a subsidiary of Merck & Co., Inc. Rahway, NJ, USA, and may hold shares and/or stock options in Merck & Co., Inc. Rahway, NJ, USA. Over the course of the study, Daniel Mines was first a full‐time employee of Merck Sharp & Dohme LLC, a subsidiary of Merck & Co., Inc. Rahway, NJ, USA (held company stock until 2020) and then of RTI Health Solutions. Suzan Esslinger, Alexis A. Krumme, and Marijo Otero‐Lobato are employees of Johnson & Johnson and hold shares and/or stock options in the company. Javier P. Gisbert has served as speaker, consultant, and advisory member for or has received research funding from MSD, AbbVie, Pfizer, Kern Pharma, Biogen, Mylan, Takeda, Janssen, Roche, Sandoz, Celgene/Bristol Myers Squibb, Gilead/Galapagos, Lilly, Ferring, Faes Farma, Shire Pharmaceuticals, Dr. Falk Pharma, Tillotts Pharma, Chiesi, Casen Fleet, Gebro Pharma, Otsuka Pharmaceutical, Norgine, and Vifor Pharma.

## Supporting information


**DATA S1.** Supporting Information.

## Data Availability

Data from the ENEIDA registry were obtained for use in this study under a licence agreement between RTI Health Solutions and GETECCU and cannot be shared with third parties.

## Appendix A

**Collaborators of the Nationwide Study on Genetic and Environmental Determinants of Inflammatory Bowel Disease (ENEIDA) Registry by the Spanish Working Group on Crohn's Disease and Ulcerative Colitis (GETECCU)**.

Beatriz Sicilia: Hospital Universitario de Burgos; David Monfort I Miquel: Consorci Sanitari de Terrassa; Xavier Calvet Calvo: Corporació Sanitària Parc Taulí de Sabadell; Miguel Mínguez Pérez: Hospital Clínico Universitario de Valencia; Luis Fernández Salazar: Hospital Clínico Universitario de Valladolid; Esther García Planella: Hospital de la Santa Creu I Sant Pau; Joaquín Hinojosa de Val: Hospital de Manises; Rufo H. Lorente Poyatos: Hospital General Universitario de Ciudad Real; Luis Bujanda: Organización Sanitaria Integrada Donostialdea; Mariana Fe Garcia Sepulcre: Hospital General Universitario de Elche; Ana Gutiérrez Casbas: Hospital General Universitario de Alicante; Eugeni Domènech: Hospital Universitari Germans Trias i Pujol; Lucía Márquez Mosquera: Hospital del Mar de Barcelona; Maria Dolores Martin Arranz: Hospital Universitario La Paz; Javier P. Gisbert: Hospital Universitario de La Princesa; Maria Esteve Comas: Hospital Universitari Mutua Terrassa; Antonio López San Roman: Hospital Universitario Ramón y Cajal; Guillermo Bastida Paz: Hospital Universitari I Politècnic La Fe; Jose Manuel Benitez Cantero: Hospital Universitario Reina Sofia; Manuel Van Domselaar: Hospital Universitario de Torrejón; Xavier Aldeguer: Hospital Universitari de Girona Dr. Josep Trueta; Fernando Bermejo: Hospital Universitario de Fuenlabrada; Jose Lazaro Perez Calle: Hospital Universitario Fundación Alcorcón; Montserrat Rivero Tirado: Hospital Universitario Marqués de Valdecilla; Santiago Garcia López: Hospital Universitario Miguel Servet; Jesús Barrio Andrés: Hospital Universitario Río Hortega; Guillermo Alcaín Martinez: Hospital Universitario Virgen de la Victoria; Elena Ricart Goméz: Hospital Clínic de Barcelona; Carla J. Gargallo Puyuelo: Hospital Clínico Universitario Lozano Blesa; Miguel Ángel Montoro Huguet: Hospital San Jorge.
