# Supplementary material for: Colectomy and Neoplasia Outcomes of Patients With Ulcerative Colitis Receiving Golimumab: A Post‐Authorisation Safety Study Using the Spanish ENEIDA Registry
Source: Pharmacoepidemiol Drug Saf. 2025 Jul 28;34(8):e70176. doi: 10.1002/pds.70176 (PMC12319186; doi:10.1002/pds.70176)
Supplement: Supplementary file 1 — DATA S1. Supporting Information. [file PDS-34-e70176-s001.docx]

# Supplementary Material

Table S-1. STROBE Checklist

|  | Item | Recommendation | Section and Notes |
| --- | --- | --- | --- |
| Title and abstract | 1 | (*a*) Indicate the study’s design with a commonly used term in the title or the abstract | The title and abstract indicate the study type. |
|  |  | (*b*) Provide in the abstract an informative and balanced summary of what was done and what was found | The abstract includes an informative and balanced summary of the study. |
| Introduction | | | |
| Background/rationale | 2 | Explain the scientific background and rationale for the investigation being reported | The rationale is discussed in the Introduction. |
| Objectives | 3 | State specific objectives, including any prespecified hypotheses | The study objectives are detailed in the Introduction and Methods. Also, as stated in the Methods, there were no prespecified hypotheses. |
| Methods | | | |
| Study design | 4 | Present key elements of study design early in the paper | Key elements of the study design are included at the top of the Methods section. |
| Setting | 5 | Describe the setting, locations, and relevant dates, including periods of recruitment, exposure, follow-up, and data collection | All relevant setting details are included in the Methods. |
| Participants | 6 | (*a*) *Cohort study*—Give the eligibility criteria, and the sources and methods of selection of participants. Describe methods of follow-up  *Case-control study*—Give the eligibility criteria, and the sources and methods of case ascertainment and control selection. Give the rationale for the choice of cases and controls | The eligibility criteria for cases and controls are summarised in the Methods and are further detailed in Table S-2. Sources and methods of selection are also described in the Methods and censoring criteria are noted in Table S-3. |
|  |  | (*b*) *Cohort study*—For matched studies, give matching criteria and number of exposed and unexposed  *Case-control study*—For matched studies, give matching criteria and the number of controls per case | Matching criteria are described in the Methods. The number of cases and controls are noted in the Results. |
| Variables | 7 | Clearly define all outcomes, exposures, predictors, potential confounders, and effect modifiers. Give diagnostic criteria, if applicable | Outcomes and exposures are described in the Methods, and further details are included in Table S-4. Potential confounders are also addressed in the Methods. |
| Data sources/ measurement | 8 | For each variable of interest, give sources of data and details of methods of assessment (measurement). Describe comparability of assessment methods if there is more than one group | Sources of data and additional assessment details are described in the Methods. |
| Bias | 9 | Describe any efforts to address potential sources of bias | Efforts to address potential confounding bias are described in the Methods and in the Discussion. |
| Study size | 10 | Explain how the study size was arrived at | Details on study size selection are included in the Methods. |
| Quantitative variables | 11 | Explain how quantitative variables were handled in the analyses. If applicable, describe which groupings were chosen and why | Variables are described in the Methods, and groupings are further depicted in Figure 1. |
| Statistical methods | 12 | (*a*) Describe all statistical methods, including those used to control for confounding | Statistical methods are detailed in the Methods section. |
|  |  | (*b*) Describe any methods used to examine subgroups and interactions | Subgroup analyses are mentioned in the Methods and are further described in the Results. |
|  |  | (*c*) Explain how missing data were addressed | As stated in the Methods, missing data were not imputed. |
|  |  | (*d*) *Cohort study*—If applicable, explain how loss to follow-up was addressed  *Case-control study*—If applicable, explain how matching of cases and controls was addressed | Follow-up and matching criteria are described in the Methods. |
|  |  | (*e*) Describe any sensitivity analyses | Sensitivity analyses are mentioned in the Methods. |
| Results | | | |
| Participants | 13 | (a) Report numbers of individuals at each stage of study—e.g. numbers potentially eligible, examined for eligibility, confirmed eligible, included in the study, completing follow-up, and analysed | Assembly of study cohorts is detailed in Table S-9. |
|  |  | (b) Give reasons for non-participation at each stage | Assembly of study cohorts is detailed in Table S-9. |
|  |  | (c) Consider use of a flow diagram | Assembly of study cohorts is detailed in Table S-9. |
| Descriptive data | 14 | (a) Give characteristics of study participants (e.g., demographic, clinical, social) and information on exposures and potential confounders | Key characteristics are noted in the Results. Further details are included in Table 1, Table S-12, and Table S-13. |
|  |  | (b) Indicate number of participants with missing data for each variable of interest | Not applicable. |
|  |  | (c) *Cohort study*—Summarise follow-up time (e.g., average and total amount) | Follow-up time is summarised in the Results and is further detailed in Table S-10. |
| Outcome data | 15 | *Cohort study*—Report numbers of outcome events or summary measures over time | Outcome data are included in the Results. |
|  |  | *Case-control study—*Report numbers in each exposure category, or summary measures of exposure | Outcome data are included in the Results. |
| Main results | 16 | (*a*) Give unadjusted estimates and, if applicable, confounder-adjusted estimates and their precision (e.g., 95% confidence interval). Make clear which confounders were adjusted for and why they were included | Crude and adjusted analyses are included in the Results. Confounders are described in Tables S-6 through S-8. |
|  |  | (*b*) Report category boundaries when continuous variables were categorised | Age groups are mentioned throughout the Results tables when relevant. |
|  |  | (*c*) If relevant, consider translating estimates of relative risk into absolute risk for a meaningful time period | Estimates of relative risk are in line with those reported in the literature, as noted in the Discussion. |
| Other analyses | 17 | Report other analyses done—e.g., analyses of subgroups and interactions, and sensitivity analyses | Subgroup and sensitivity analyses are mentioned in the Results. |
| Discussion | | | |
| Key results | 18 | Summarise key results with reference to study objectives | Key results are detailed throughout the Discussion and are reiterated in the Conclusions. |
| Limitations | 19 | Discuss limitations of the study, taking into account sources of potential bias or imprecision. Discuss both direction and magnitude of any potential bias | Limitations are addressed in the Discussion. |
| Interpretation | 20 | Give a cautious overall interpretation of results considering objectives, limitations, multiplicity of analyses, results from similar studies, and other relevant evidence | Cautious interpretations are included throughout the Discussion. |
| Generalisability | 21 | Discuss the generalisability (external validity) of the study results | Generalisability is mentioned in the Discussion. |
| Other information | | | |
| Funding | 22 | Give the source of funding and the role of the funders for the present study and, if applicable, for the original study on which the present article is based | Sources of funding and authors’ conflicts of interest are included in the manuscript’s disclosure section. |

Table S-2. Identification of Patients for Each Analysis

| Identification of Patients for the Cohort Analyses |
| --- |
| Patients entered the cohort study population if they met the following criteria:   - Were from a research-quality site that contributed data to the ENEIDA registry - Were aged 18 years or older at the date of study therapy initiation - Had a diagnosis of UC - Initiated therapy with GLM, an anti-TNFα agent other than GLM, or TP starting 19 September 2013 (the date of GLM EU approval for UC) through 31 December 2021 - Date of first prescription of cohort-defining therapy (index date) occurred within a clinically credible period (< 6 months) after the last recorded (i.e., most recent) clinic visit in ENEIDA (dates of some index prescriptions do not have corresponding clinic visits); index dates beyond this range raise concerns that the clinical record for this patient may be incomplete |
| Patients were excluded if, before cohort entry:   - They had experienced any of the study outcomes of interest (underwent partial or complete colectomy or received a diagnosis of ACN or HSTCL) before or on the cohort entry date - For each of the 3 study cohorts (i.e., GLM, other anti-TNFα agents, and TPs), the patient initiated the cohort-defining therapy before 19 September 2013 (i.e., they were prevalent users of that therapy at the study start date). However, patients could enter the study later based on subsequent initiation of other cohort-defining therapies - Patients had initiated a novel biological or immunomodulator agent before cohort entry (i.e., vedolizumab, natalizumab, visilizumab, denosumab, etrolizumab, tocilizumab, ustekinumab, certolizumab, tofacitinib)^a^ - The indication for which a study therapy was prescribed was not UC |
| Identification of Cases and Controls for the Nested Case-Control Analyses |
| Controls were eligible if, on the reference date, they:   - Were alive, were under observation, and had not yet experienced the outcome - Had been exposed to a relevant study therapy within the applicable risk window leading to the reference date (i.e., within 90 days prior to the reference date for colectomy; any date prior to the reference date for ACN or CRC outcomes) - Had a duration of UC similar to that of the corresponding case (i.e., within ± 12 months) - Had a length of follow-up time in the study similar to that of the corresponding case (i.e., within ± 3 months) |

ACN = advanced colonic neoplasia; CRC = colorectal cancer; ENEIDA = Nationwide Study on Genetic and Environmental Determinants of Inflammatory Bowel Disease (Spain); EU = European Union; GLM = golimumab; HSTCL = hepatosplenic T-cell lymphoma; TNFα = tumour necrosis factor α; TP = thiopurine; UC = ulcerative colitis.

^a^ This list includes therapies that would likely be prescribed to patients with more severe UC and that are potentially related to the study outcomes; it also includes therapies becoming newly available to treat UC or its complications.

Table S-3. Censoring Criteria

| Criteria Common to All Analyses |
| --- |
| - Withdrawal from the registry - Death - End of study period (30 March 2022) - Loss of follow-up: based on common clinical practice in Spain, patients receiving anti-TNFα agents or immunomodulatory therapies would be expected to have regular follow-up visits at least every 6 months. Patients who appeared to be exposed to study therapies (as indicated by date of therapy prescription or dispensing) but who had no recorded follow-up visit for at least 13 months after the last clinical contact (i.e., missed 2 follow-up visits) were deemed “lost to follow-up” and censored 6 months after the last follow-up visit. - Initiation of a novel immunomodulatory therapy that was newly marketed during the study period: these agents may have a direct effect on study outcomes and are likely to be preferentially prescribed to patients with UC with more active or severe disease. Therefore, including person-time exposed to these therapies would have very likely led to intractable confounding and uninterpretable results. Because uncontrolled disease activity may prompt therapeutic changes, it is possible that censoring follow-up immediately after switching may mask effects of the therapy that preceded the switch. For this reason, the date of censoring was set 90 days after the start of the novel immunomodulatory therapy. - Prescription of 2 anti-TNFα agents on the same date |
| Criteria Specifically for Analysis of Colectomy Due to Intractable Disease |
| - Colectomy due to intractable disease - Total colectomy due to any cause - Other types of colectomy, depending on reason for operation, patient characteristics, and timing of any subsequent colectomy - Diagnosis of ACN or CRC: these conditions are commonly treated by colectomy (total or partial). According to the study principal investigators (clinical specialists in UC), the amount of residual colon left after a partial colectomy is small enough that the risk of a second colectomy due to intractable UC is greatly reduced. Therefore, the risk in patients who have had a partial colectomy is no longer comparable to the risk among patients with a complete colon. Patients who experience ACN/CRC but who do not have a colectomy performed are most likely too sick to undergo colectomy, in which case, these patients are also unlikely to be at risk for colectomy due to intractable disease. |
| Criteria Specifically for Analysis of ACN and CRC |
| - Occurrence of ACN or CRC - Total or partial colectomy for any reason: Patients who have undergone a total colectomy have virtually no tissue left in which ACN can develop. The amount of residual colon left after a partial colectomy is small enough that the risk of developing ACN is greatly reduced. Therefore, the risk in patients who have had a partial colectomy is no longer comparable to the risk among patients with a complete colon. |
| Criteria Specifically for Analysis of HSTCL |
| - The occurrence of HSTCL censored follow-up for further diagnoses of HSTCL |

ACN = advanced colonic neoplasia; CRC = colorectal cancer; HSTCL = hepatosplenic T-cell lymphoma; TNFα = tumour necrosis factor α; UC = ulcerative colitis.

Table S-4. Definitions of Study Outcomes and Cohort Analysis Methods

| Outcome Definitions |
| --- |
| Total or Partial Colectomy Due to Intractable Disease   - Information about bowel surgery is considered mandatory data in ENEIDA. Categories available include: “intractable disease” (“refractoriness to medical treatment”), “stenosis,” “perforation,” “hemorrhage,” “dysplasia or cancer,” and “other.” The date of colectomy is also available in the ENEIDA database. Based on consultation with the study principal investigators (clinical specialists in IBD) and clinical guidelines,^1^ the following scenarios were deemed to meet the definition of colectomy due to intractable disease when no other reasons for colectomy were indicated: - Subtotal colectomy with terminal ileostomy among patients aged 65 years or older (i.e., elderly) - Subtotal colectomy with ileorectal anastomosis among women aged up to 50 years (i.e., women of childbearing age) - In these 2 specific subpopulations (elderly and women of childbearing age), the conservative approach consisting of a partial colectomy is the currently preferred surgical approach to treat resistant UC   ACN (a composite endpoint that includes both CRC and HGD)   - The rationale for using a composite endpoint was that both constituents represent different phases of the same disease pathway. Current understanding of CRC is that all such cancers go through a dysplastic phase before malignant transformation. Patients with HGD have a high probability of progressing to CRC, and like patients with CRC, they are typically treated with colectomy.^2^   HSTCL   - Information about HSTCL was ascertained from the ENEIDA registry. However, cases of HSTCL may be difficult to capture because these rare lymphoid malignancies can conceivably be classified as lymphomas without further specification in the ENEIDA registry. To ensure that all reported cases of HSTCL in the ENEIDA registry were captured, registry data managers performed a sensitive search for lymphoma-related diagnoses in the registry free-text fields on a yearly basis to identify potential cases. Any identified patients were further evaluated by the investigators at the corresponding hospitals to confirm or rule out a diagnosis of HSTCL. |
| Cohort Analysis Methods |
| - In time-to-event analyses (e.g., Cox regression for calculating HRs), patients must have been followed until the occurrence of the study outcome or a censoring event, thereby incorporating any periods of non-exposure to study therapies. However, in Poisson regression analyses (for calculating IRRs), follow-up time while exposed was the main unit of analysis. As such, for the outcome of colectomy due to intractable disease, a univariable Poisson regression model with log-time offset was generated in which the occurrence of the outcome was modelled only as a function of study exposure category (treated as a multilevel categorical variable). Additionally, a univariable Cox regression model was generated in which all possible categories of exposure to the therapies of interest were considered. For both models, exposure category was treated as a time-varying variable for all outcome analyses, when applicable. Cox regression models were also generated for the outcomes of ACN and CRC, but not for HSTCL, due to the absence of any events observed. |

ACN = advanced colonic neoplasia; CRC = colorectal cancer; ENEIDA = Nationwide Study on Genetic and Environmental Determinants of Inflammatory Bowel Disease (Spain); HGD = high-grade colorectal dysplasia; HR = hazard ratio; HSTCL = hepatosplenic T-cell lymphoma; IBD = inflammatory bowel disease; IRR = incidence rate ratio; UC = ulcerative colitis.

Note: Only incident diagnoses (those with an onset after cohort entry) qualified as outcomes.

Table S-5. Agreement of ENEIDA Data With Medical Chart Data

| Summary | |
| --- | --- |
| - To substantiate the validity of using the abstracted information for the stated study objectives, an exploratory analysis was conducted to examine the agreement between data elements from the ENEIDA registry and data on variables obtained from chart abstraction. - Although there were some differences in the recording of data between the ENEIDA registry and the medical charts, data were reasonably well aligned for the variables evaluated. Data from 2 key variables (listed below) suggested that when ± 30 days of discrepancy were allowed, ≥ 95% of ENEIDA registry dates were concordant with the corresponding medical chart dates. | |
| Agreement on date of first golimumab use^a^ | % |
| Same date | 23.5 |
| Same date +/- 1 month | 100 |
| Disagreement of more than +/- 1 month | 0 |
| Agreement on date of colectomy ^b^ | % |
| Same date | 31.0 |
| Same date +/- 1 month | 95.2 |
| Disagreement of more than +/- 1 month | 4.8 |

ENEIDA = Nationwide Study on Genetic and Environmental Determinants of Inflammatory Bowel Disease (Spain); NCC = nested case-control; UC = ulcerative colitis.

Note: Percentages of agreement are based on the comparison of information on each selected variable coming from routine ENEIDA data with the corresponding data abstracted from the medical charts of cases and controls in the NCC analyses.

^a^ Comparison among 34 patients with nonmissing data in both source documents.

^b^ Comparison among 42 patients with nonmissing data in both source documents.

Table S-6. Candidate Variable Screening and Selection Methods

| Cohort analyses |
| --- |
| Screening  For adjusted models, only variables associated with the outcome, regardless of association with exposure, were included to reduce the probability of having instrumental variables. To identify which covariates to consider in the multivariable models, separate univariate regression models were generated for each candidate variable of interest (Table S-7). To be considered for inclusion, variables needed to exhibit an IRR greater than 1.25, or less than its inverse (i.e., 0.80). Moreover, other variables deemed to be clinically important by the research team (i.e., sex) were also included in the model. If the number of variables that qualified on the basis of these cutoffs was larger than could be supported in the multivariable adjustment model (i.e., the model would not converge), additional considerations—such as strength of association in the univariate model and perceived clinical importance—were applied to build a more parsimonious model.**^3^**  Selection  In the cohort analyses, 3 clinically relevant variables were selected that satisfied the statistical screening threshold (age group, ulcerative colitis duration, and prior treatment with cyclosporine [which may be considered a proxy for treatment-resistant disease]^4^). Sex was also included as a variable, as it was deemed clinically relevant despite its association not meeting the screening threshold criteria. |
| NCC analyses |
| Screening  The ascertainment window for covariates of interest for cases and controls was the 1-year period before starting the most recent study therapy. As with the variable screening process in the cohort analyses, a separate conditional logistic regression model was generated for each candidate variable of interest (Table S-8).  Selection  NCC analyses were adjusted for age, any hospitalisation (due to significant association), prior treatment with cyclosporine, and sex. |

IRR = incidence rate ratio; NCC = nested case-control.

Note: The number of candidate variables that qualified for consideration in the multivariable adjustment models was greater than could be accommodated given the limited number of outcomes observed. The validity of the Cox models was confirmed by inspecting the proportionality of the hazards, and the validity of the Poisson models was confirmed by inspecting the overdispersion and goodness of fit.

Table S-7. Candidate Variable Screening for Cohort Analyses

| Candidate variable | Colectomy due to intractable disease | | ACN |
| --- | --- | --- | --- |
|  | Poisson model,  IRR (95% CI) | Cox model,  HR (95% CI) | Cox model,  HR (95% CI) |
| Age group |  |  |  |
| 35 to < 65 years vs. 18 to < 35 years | 1.10 (0.57-2.11) | 0.94 (0.52-1.67) | 1.70 (0.35-8.19) |
| ≥ 65 years vs. 18 to < 35 years | 2.32 (0.97-5.54) | 2.12 (1.01-4.45) | 5.54 (0.93-33.19) |
| Sex (male vs. female) | 1.56 (0.89-2.73) | 1.37 (0.83-2.26) | 1.73 (0.52-5.76) |
| Calendar year of cohort entry |  |  |  |
| 2013-2015 vs. 2019-2021 | 0.65 (0.32-1.34) | 1.59 (0.82-3.05) | 0.89 (0.09-8.47) |
| 2016-2018 vs. 2019-2021 | 0.72 (0.35-1.48) | 1.16 (0.60-2.27) | 1.05 (0.11-9.90) |
| UC duration in years |  |  |  |
| ≥ 67th percentile vs. < 33rd percentile | 0.50 (0.26-0.95) ^a^ | 0.55 (0.31-0.99) ^a^ | 1.97 (0.36-10.78) ^b^ |
| 33rd to < 67th percentile vs. < 33rd percentile | 0.48 (0.24-0.93) ^a^ | 0.45 (0.24-0.84) ^a^ | 2.79 (0.56-13.80) ^b^ |
| Maximum extent of disease |  |  |  |
| Extensive vs. left side only | 1.41 (0.78-2.57) | 1.61 (0.92-2.81) | 0.76 (0.22-2.61) |
| Proctitis vs. left side only | 0.39 (0.05-2.95) | 0.70 (0.16-3.00) | NE |
| Not recorded vs. left side only | 1.86 (0.61-5.63) | 1.60 (0.54-4.72) | 3.43 (0.67-17.68) |
| Prior treatment with steroids (yes vs. no) | 1.38 (0.77-2.47) | 1.56 (0.90-2.69) | 0.46 (0.15-1.45) |
| Prior treatment with cyclosporine (yes vs. no) | 3.46 (1.72-6.98) | 3.66 (1.99-6.73) | 5.12 (1.38-18.91) |
| Hospitalised for UC (yes vs. no) | 1.44 (0.79-2.63) | 1.27 (0.73-2.21) | 0.82 (0.18-3.76) |
| Prior diagnosis with PSC (yes vs. no) | 0.50 (0.21-1.19) | NE | NE |
| Prior screening colonoscopy (yes vs. no) | NE | 0.62 (0.30-1.31) | 3.06 (0.97-9.66) |
| Number of previous anti-TNFα agents |  |  |  |
| 1 vs. 0 | NE | 2.14 (1.16-3.96) | NE |
| 2 vs. 0 | NE | NE | NE |
| Recent switcher after use of another anti-TNFα agent^c^ |  |  |  |
| After short-term use (≤ 3 months) vs. did not switch | NE | 3.74 (0.51-27.51) | N/A |
| After long-term use (> 3 months) vs. did not switch | 6.04 (1.47-24.86) | 6.71 (1.60-28.26) | N/A |

ACN = advanced colorectal neoplasia; CI = confidence interval; HR = hazard ratio; IRR = incidence rate ratio; N/A = not applicable; NE = not estimable; PSC = primary sclerosing cholangitis; TNFα = tumour necrosis factor α; UC = ulcerative colitis.

Note: Each row represents a separate univariable model assessing the association of each candidate variable with the outcome of interest. Incidence rate ratios and 95% CIs were derived from Poisson regression models with log-time offset. Hazard ratios and 95% CIs were derived from a Cox regression model.

^a^ Duration of UC was continually updated. At the start of the treatment (i.e., at baseline), the 33rd percentile for UC duration was 2.0 years and the 67th percentile was 8.6 years.

^b^ UC duration was continually updated at any change in a patient’s exposure category. At baseline, across the population included in this analysis, the 33rd percentile for UC duration was 1.5 years and the 67th percentile was 7.5 years.

^c^ Recent switcher refers to a patient who started a new anti-TNFα agent within 90 days of discontinuing another anti-TNFα agent.

Table S-8. Candidate Variable Screening for NCC

| Candidate variable | Colectomy due to intractable disease,  OR (95% CI) |
| --- | --- |
| Age group |  |
| ≥ 35 years and < 65 years vs. 18 to < 35 years | 1.28 (0.50-3.32) |
| ≥ 65 years vs. 18 to < 35 years | 2.10 (0.61-7.24) |
| Sex (male vs. female) | 1.54 (0.67-3.54) |
| UC duration in years^a^ |  |
| ≥ 67th percentile vs. < 33rd percentile | NE |
| 33rd to < 67th percentile vs. < 33rd percentile | NE |
| Maximum extent of disease period 1^b^ |  |
| Proctitis vs. extensive | 1.57 (0.39-6.36) |
| Left side only vs. extensive | 1.23 (0.38-3.95) |
| Not recorded vs. extensive | NE |
| Maximum extent of disease period 2^b^ |  |
| Proctitis vs. extensive | 0.72 (0.20-2.55) |
| Left side only vs. extensive | 0.39 (0.13-1.13) |
| Not recorded vs. extensive | 1.08 (0.15-7.75) |
| Prior treatment with steroids period 1^b^ (yes vs. no) | 1.09 (0.40-2.97) |
| Prior treatment with steroids period 2^b^ (yes vs. no) | 0.67 (0.26-1.72) |
| Prior treatment with cyclosporine period^b^ 1 (yes vs. no) | NE |
| Prior treatment with cyclosporine period 2 ^b^ (yes vs. no) | 0.14 (0.03-0.65) |
| Number of hospitalisations for UC period 1^b^ |  |
| 1 or 2 vs. 0 | 2.30 (0.86-6.16) |
| ≥ 3 vs. 0 | 1.35 (0.19-9.45) |
| Number of hospitalisations for UC period 2^b^ |  |
| 1 or 2 vs. 0 | 3.88 (1.52-9.92) |
| ≥ 3 vs. 0 | 1.63 (0.15-17.63) |
| Prior diagnosis with PSC (yes vs. no) | NE |
| Prior screening colonoscopy (yes vs. no) | 0.66 (0.23-1.89) |
| Number of previous anti-TNFα agents |  |
| 1 vs. 0 | NE |
| 2 vs. 0 | NE |
| 3 or more vs. 0 | NE |
| Recent switcher after use of another anti-TNFα agent^c^ |  |
| After short-term use (≤ 3 months) vs. did not switch | 0.88 (0.30-2.63) |
| After long-term use (> 3 months) vs. did not switch | 1.18 (0.25-5.53) |

CI = confidence interval; NCC = nested case-control; NE = not estimable; OR = odds ratio; PSC = primary sclerosing cholangitis; TNFα = tumour necrosis factor α; UC = ulcerative colitis.

Note: Each row represents a separate univariable regression model assessing the association of each candidate variable with the outcome. ORs and 95% CI were derived from conditional logistic regression models.

^a^ UC duration was ascertained on the date of the event or the corresponding reference date for control patients. The 33rd percentile was 2.6 years, and the 67th percentile was 8.3 years.

^b^ Period 1: the first year after UC diagnosis, or until cohort entry (whichever occurred first). Period 2: the year before the last episode of a study therapy commenced, looking backward from the date of study outcome (or an equivalent date among the controls)—this period could extend back only as far as the date of the first UC diagnosis and thus could be truncated.

^c^ Recent switcher refers to a patient who started a new anti-TNFα agent within 90 days of discontinuing another anti-TNFα agent.

Table S-9. Assembly of Study Cohorts

| Entry criterion | Study cohort | | |
| --- | --- | --- | --- |
|  | GLM | Other anti-TNFα agents | TP |
| Diagnosis of UC from a qualified site | 19,738 | 19,738 | 19,738 |
| AND Initiated study therapy for first time on or after 19 September 2013 | 546 | 2,021 | 1,914 |
| AND Age 18 years or older at study therapy initiation | 540 | 1,932 | 1,792 |
| AND Study therapy initiated within a credible period (< 6 months) after last ENEIDA clinic visit^a^ | 518 | 1,864 | 1,751 |
| AND No previous exposure to any other anti-TNFα before cohort entry^b^ | N/A | N/A | 1,396 |
| AND No previous exposure to vedolizumab or other novel biologic agents^c^ before cohort entry | 488 | 1,813 | 1,393 |
| AND No partial or complete colectomy before cohort entry | 477 | 1,744 | 1,381 |
| AND No ACN before cohort entry | 477 | 1,740 | 1,380 |
| AND No HSTCL before cohort entry | 477 | 1,740 | 1,380^e^ |
| AND No double prescription of anti-TNFα, GLM, or vedolizumab or similar novel biologic agents^d^ | 474^e^ | 1,737^e^ | N/A |

ACN = advanced colonic neoplasia; ENEIDA = Nationwide Study on Genetic and Environmental Determinants of Inflammatory Bowel Disease (Spain); GLM = golimumab; HSTCL = hepatosplenic T-cell lymphoma; N/A = not applicable; TNFα = tumour necrosis factor α; TP = thiopurine; UC = ulcerative colitis.

Note: Study cohorts are not mutually exclusive. Patients can qualify for >1 cohort if they meet all applicable criteria.

^a^ Dates of some initial study therapy prescriptions did not have corresponding clinic visits (for GLM, n=22; for anti-TNFα agents, n=68; for TP, n=41).

^b^ Criterion to be applied to the thiopurine cohort only; will not affect numbers for GLM or other anti-TNFα agent cohorts.

^c^ Agents include the following: vedolizumab, natalizumab, etrolizumab, tocilizumab, ustekinumab, certolizumab, etanercept, or any similar newly marketed therapy.

^d^ Overall, 3 patients were excluded for having a prescription for 2 different anti-TNFα agents on the same date. Because co-prescription of >1 anti-TNFα agent is highly atypical, these patients were excluded out of concern about their data quality.

^e^ Final analytical cohort.

Table S-10. Duration of Follow-up by Cohort

| Duration of follow-up by cohort for the colectomy outcome | | | |
| --- | --- | --- | --- |
|  | GLM^a^  (N = 471) | Other anti-TNFα agents ^a^ (N = 1,734) |  |
| Months of follow-up^b^ |  |  |  |
| Mean (SD) | 23 (23.5) | 26 (24.0) |  |
| Median (Q1, Q3) | 13 (5.9, 32.9) | 17 (7.6, 38.4) |  |
| Min, max | 0.0, 94.1 | 0.0, 101.8 |  |
| Duration of follow-up by cohort for the ACN outcome | | | |
|  | GLM  (N = 474) | Other anti-TNFα agents  (N = 1,737) | TP  (N = 1,380) |
| Months of follow-up^c^ |  |  |  |
| Mean (SD) | 34 (26.1) | 32 (26.3) | 34 (26.6) |
| Median (Q1, Q3) | 27 (12.0, 51.7) | 23 (9.9, 51.3) | 25 (9.9, 54.1) |
| Min, max | 0.8, 95.0 | 0.1, 101.9 | 0.6, 102.3 |
| Duration of follow-up by cohort for the HSTCL outcome | | | |
|  | GLM  (N = 474) | Other anti-TNFα agents  (N = 1,737) | TP  (N = 1,380) |
| Months of follow-up^c^ |  |  |  |
| Mean (SD) | 35 (26.5) | 33 (26.5) | 34 (26.7) |
| Median (Q1, Q3) | 28 (13.0, 52.9) | 25 (10.7, 52.7) | 25 (9.9, 54.7) |
| Min, max | 0.8, 95.0 | 0.4, 101.9 | 0.6, 102.3 |

ACN = advanced colonic neoplasia; GLM = golimumab; HSTCL = hepatosplenic T-cell lymphoma; Q1 = first quartile; Q3 = third quartile; SD = standard deviation; TNFα = tumour necrosis factor α; TP = thiopurine.

Note: Study cohorts were not mutually exclusive. Patients could qualify for >1 cohort if they met all applicable criteria.

^a^ This table includes only those patients who contributed at least some person-time to the GLM-only exposure category or to the other anti-TNFα agent–only exposure category after cohort entry. This table does not include the follow-up time of 6 patients who, during all follow-up time, were exposed exclusively to GLM in combination with other anti-TNFα agents (3 patients in the GLM cohort and 3 patients in the other anti-TNFα agent cohort), although that person-time is included in the comparative analyses in the overlapping (i.e., combined) exposure category.

^b^ Follow-up for the colectomy analyses includes a 90-day extension period of the risk window after discontinuation (i.e., stop date) of the evaluated treatments.

^c^ Follow-up for ACN and HSTCL used an “ever exposed, always at risk” approach.

Table S-11. Reasons for End of Follow-up by Cohort

| Reasons for end of follow-up for all outcomes except HSTCL, 2013-2021 | | | |
| --- | --- | --- | --- |
|  | GLM  (N = 474) | Other anti-TNFα agents  (N = 1,737) | TP  (N = 1,380) |
| Withdrawal from registry, n (%) | 12 (2.5) | 47 (2.7) | 50 (3.6) |
| Death, n (%) | 2 (0.4) | 11 (0.6) | 14 (1.0) |
| End of study period, n (%) | 129 (27.2) | 459 (26.4) | 368 (26.7) |
| Total or partial colectomy for any cause, n (%) | 11 (2.3) | 60 (3.5) | 18 (1.3) |
| ACN or CRC diagnosis, n (%) | 2 (0.4) | 6 (0.3) | 4 (0.3) |
| Loss of follow-up , n (%)^a^ | 164 (34.6) | 647 (37.3) | 690 (50.0) |
| Initiation of vedolizumab or other novel biologic agents, n (%) | 154 (32.5) | 507 (29.2) | 235 (17.0) |
| Prescription of 2 anti-TNFα agents on the same date, n (%) | 0 (0.0) | 0 (0.0) | 1 (0.1) |
| Reasons for end of follow-up for HSTCL, 2013-2021 | | | |
|  | GLM  (N = 474) | Other anti-TNFα agents  (N = 1,737) | TP  (N = 1,380) |
| Withdrawal from registry, n (%) | 14 (3.0) | 54 (3.1) | 52 (3.8) |
| Death, n (%) | 2 (0.4) | 13 (0.7) | 14 (1.0) |
| End of study period, n (%) | 136 (28.7) | 469 (27.0) | 375 (27.2) |
| Loss of follow-up, n (%)^a^ | 165 (34.8) | 676 (38.9) | 698 (50.6) |
| Initiation of vedolizumab or other novel biologic agents, n (%) | 157 (33.1) | 525 (30.2) | 240 (17.4) |
| Prescription of 2 anti-TNFα agents on the same date, n (%) | 0 (0.00) | 0 (0.00) | 1 (0.1) |

ACN = advanced colonic neoplasia; CRC = colorectal cancer; GLM = golimumab; HSTCL = hepatosplenic T-cell lymphoma; N/A = not applicable; Q1 = first quartile; Q3 = third quartile; SD = standard deviation; TNFα = tumour necrosis factor α; TP = thiopurine.

Note: Study cohorts were not mutually exclusive. Patients could qualify for >1 cohort if they met all applicable criteria.

^a^ Patients who had no recorded follow-up visit for at least 13 months after the last clinical contact (i.e., missed 2 follow-up visits) were deemed “lost to follow-up” and censored at 6 months after the last follow-up visit.

Table S-12. Description of Cases and Controls at the Reference Date for NCC Analyses

|  | Colectomy due to intractable disease | | Advanced colorectal neoplasia | |
| --- | --- | --- | --- | --- |
|  | Cases (N = 41) | Controls (N = 70) | Cases (N = 9) | Controls (N = 11) |
| Exposure category, n (%) |  |  |  |  |
| GLM only | 3 (7.3) | 19 (27.1) | 1 (11.1) | 2 (18.2) |
| Other anti-TNFα agents only | 35 (85.4) | 49 (70.0) | 5 (55.6) | 6 (54.5) |
| GLM + other anti-TNFα agents | 3 (7.3) | 2 (2.9) | N/A | N/A |
| TP | N/A | N/A | 3 (33.3) | 3 (27.3) |
| Age (years) |  |  |  |  |
| n | 41 | 70 | 9 | 11 |
| Mean (SD) | 49.7 (18.2) | 46.3 (17.1) | 58.2 (16.67) | 47.2 (19.21) |
| Median (Q1, Q3) | 52.0 (35.0, 64.0) | 47.0 (33.0, 57.0) | 66.0 (62.0, 67.0) | 49.0 (28.0, 65.0) |
| Min, Max | 19.0, 82.0 | 19.0, 84.0 | 27.0, 74.0 | 24.0, 84.0 |
| Age group, n (%) |  |  |  |  |
| 18 to < 35 years | 10 (24.4) | 22 (31.4) | 2 (22.2) | 4 (36.4) |
| 35 to < 65 years | 21 (51.2) | 37 (52.9) | 2 (22.2) | 4 (36.4) |
| ≥ 65 years | 10 (24.4) | 11 (15.7) | 5 (55.6) | 3 (27.3) |
| Sex, n (%) |  |  |  |  |
| Female | 13 (31.7) | 31 (44.3) | 4 (44.4) | 3 (27.3) |
| Male | 28 (68.3) | 39 (55.7) | 5 (55.6) | 8 (72.7) |
| Calendar year of reference date, n (%) |  |  |  |  |
| 2013 | 2 (4.9) | 3 (4.3) | 0 (0) | 0 (0) |
| 2014 | 6 (14.6) | 11 (15.7) | 0 (0) | 0 (0) |
| 2015 | 8 (19.5) | 14 (20.0) | 1 (11.1) | 2 (18.2) |
| 2016 | 12 (29.3) | 22 (31.4) | 0 (0) | 0 (0) |
| 2017 | 8 (19.5) | 12 (17.1) | 1 (11.1) | 1 (9.1) |
| 2018 | 3 (7.3) | 5 (7.1) | 7 (77.8) | 8 (72.7) |
| 2019 | 2 (4.9) | 3 (4.3) | 0 (0) | 0 (0) |
| 2020 | 0 (0) | 0 (0) | 0 (0) | 0 (0) |
| 2021 | 0 (0) | 0 (0) | 0 (0) | 0 (0) |
| UC duration in years |  |  |  |  |
| n | 41 | 69 | 8 | 11 |
| Mean (SD) | 7.9 (9.3) | 7.2 (8.8) | 12.5 (9.78) | 10.4 (6.72) |
| Median (Q1, Q3) | 4.6 (1.0,10.6) | 4.4 (1.1,9.3) | 9.7 (4.4, 20.9) | 8.7 (6.5, 12.2) |
| Min, Max | 0.0, 38.9 | 0.1, 43.0 | 3.3, 27.1 | 3.4, 28.0 |
| Maximum extent of disease period 1, n (%)^a^ |  |  |  |  |
| Extensive | 14 (43.8) | 26 (47.3) | 2 (40.0) | 1 (11.1) |
| Left sided only | 12 (37.5) | 18 (32.7) | 1 (20.0) | 2 (22.2) |
| Other | 0 (0.0) | 3 (5.5) | 0 (0.0) | 2 (22.2) |
| Proctitis | 6 (18.8) | 8 (14.5) | 2 (40.0) | 4 (44.4) |
| Maximum extent of disease period 2, n (%) ^a^ |  |  |  |  |
| Extensive | 22 (56.4) | 23 (41.8) | 2 (33.3) | 5 (45.5) |
| Left sided only | 9 (23.1) | 22 (40.0) | 1 (16.7) | 3 (27.3) |
| Other | 2 (5.1) | 2 (3.6) | 0 (0.0) | 2 (18.2) |
| Proctitis | 6 (15.4) | 8 (14.5) | 3 (50.0) | 1 (9.1) |
| Treatment with steroids period 1, n (%) ^a^ |  |  |  |  |
| No | 12 (29.3) | 21 (30.0) | 4 (44.4) | 4 (36.4) |
| Yes | 29 (70.7) | 49 (70.0) | 5 (55.6) | 7 (63.6) |
| Treatment with steroids period 2, n (%) ^a^ |  |  |  |  |
| No | 8 (19.5) | 19 (27.1) | 4 (44.4) | 8 (72.7) |
| Yes | 33 (80.5) | 51 (72.9) | 5 (55.6) | 3 (27.3) |
| Treatment with cyclosporine period 1, n (%) ^a^ |  |  |  |  |
| No | 35 (87.5) | 68 (100.0) | 5 (71.4) | 11 (100.0) |
| Yes | 5 (12.5) | 0 (0.0) | 2 (28.6) | 0 (0.0) |
| Treatment with cyclosporine period 2, n (%) ^a^ |  |  |  |  |
| No | 32 (78.0) | 65 (97.0) | 5 (71.4) | 11 (100.0) |
| Yes | 9 (22.0) | 2 (3.0) | 2 (28.6) | 0 (0.0) |
| Number of hospitalisations for UC period 1, n (%) ^a^ |  |  |  |  |
| 0 | 17 (48.6) | 43 (67.2) | 5 (71.4) | 7 (70.0) |
| 1 or 2 | 16 (45.7) | 17 (26.6) | 2 (28.6) | 3 (30.0) |
| 3 or more | 2 (5.7) | 4 (6.3) |  |  |
| Number of hospitalisations for UC period 2, n (%) ^a^ |  |  |  |  |
| 0 | 12 (30.0) | 40 (58.8) | 5 (71.4) | 9 (81.8) |
| 1 or 2 | 27 (67.5) | 25 (36.8) | 2 (28.6) | 2 (18.2) |
| 3 or more | 1 (2.5) | 3 (4.4) | 0 (0) | 0 (0) |
| Prior diagnosis with PSC, n (%) |  |  |  |  |
| No | 41 (100.0) | 68 (97.1) | 9 (100) | 11 (100) |
| Yes | 0 (0) | 2 (2.9) | 0 (0) | 0 (0) |
| Prior screening colonoscopy period 2, n (%)^a^ |  |  |  |  |
| No | 28 (71.8) | 44 (67.7) | 3 (42.9) | 8 (72.7) |
| Yes | 11 (28.2) | 21 (32.3) | 4 (57.1) | 3 (27.3) |
| Number of previous anti-TNFα agents, n (%) |  |  |  |  |
| 0 | 0 (0) | 4 (5.9) | 1 (12.5) | 2 (18.2) |
| 1-2 | 39 (95.1) | 59 (86.8) | 5 (62.5) | 7 (63.6) |
| 3 or more | 2 (4.9) | 5 (7.4) | 2 (25.0) | 2 (18.2) |
| Recent switcher after use of another anti-TNFα agent, n (%)^b^ |  |  |  |  |
| After short-term use  (≤ 3 months) | 6 (14.6) | 12 (17.1) | 3 (75.0) | 1 (11.1) |
| After long-term use  (> 3 months) | 3 (7.3) | 4 (5.7) | 0 (0) | 1 (11.1) |
| Did not switch | 32 (78.0) | 54 (77.1) | 1 (25.0) | 7 (77.8) |

GLM = golimumab; NCC = nested case-control; PSC = primary sclerosing cholangitis; Q1 = first quartile; Q3 = third quartile; SD = standard deviation; TNFα = tumour necrosis factor α; TP = thiopurines; UC = ulcerative colitis.

Notes: The reference date for a case was the date that the patient experienced the outcome, while the reference date for a control was the date that their corresponding case experienced the outcome. For the colectomy due to intractable disease outcome, TP was not considered an exposure per the protocol. For the colectomy outcome, the exposure period of interest was anytime within 90 days before the reference date. For neoplasia outcomes, the exposure period of interest was any time before the reference date. Information on some variables sought in chart review was not always documented in the medical record. In such situations, the sum of patients across all categories of that variable may be less than the sum of case and control patients.

^a^ Period 1: the first year after UC diagnosis, or until cohort entry (whichever occurred first). Period 2: the year before the last episode of a study therapy commenced, looking backward from the date of study outcome (or an equivalent date among the controls)—this period could extend back only as far as the date of the first UC diagnosis and thus could be truncated.

^b^ Recent switcher refers to a patient who started a new anti-TNFα agent within 90 days of discontinuing another anti-TNFα agent.

Table S-13. Characteristics of Those With Overlapping Exposure to Both GLM and Other anti-TNFα Therapies

| Characteristic | Overlapping exposure to both GLM and other anti-TNFα therapies |
| --- | --- |
| Age in years |  |
| n | 185 |
| Mean (SD) | 45.3 (14.85) |
| Median (Q1, Q3) | 45.0 (34.0, 56.0) |
| Min, Max | 18.0, 87.0 |
| Age group, n (%) |  |
| 18 to <35 years | 54 (29.2) |
| 35 to <65 years | 112 (60.5) |
| ≥65 years | 19 (10.3) |
| Sex, n (%) |  |
| Male | 92 (49.7) |
| Female | 93 (50.3) |
| Calendar year of study entry, n (%) |  |
| 2013 | 0 (0) |
| 2014 | 17 (9.2) |
| 2015 | 27 (14.6) |
| 2016 | 34 (18.4) |
| 2017 | 34 (18.4) |
| 2018 | 27 (14.6) |
| 2019 | 18 (9.7) |
| 2020 | 15 (8.1) |
| 2021 | 13 (7.0) |
| UC duration in years |  |
| n | 185 |
| Mean (SD) | 8.7 (7.85) |
| Median (Q1, Q3) | 6.2 (2.3, 12.8) |
| Min, max | 0.1, 36.3 |
| Maximum extent of disease, n (%)^a^ |  |
| Extensive | 89 (48.1) |
| Left side only | 74 (40.0) |
| Proctitis | 14 (7.6) |
| Extent not defined | 8 (4.3) |
| Prior treatment with steroids, n (%) |  |
| No | 67 (36.2) |
| Yes | 118 (63.8) |
| Prior treatment with cyclosporine, n (%) |  |
| No | 178 (96.2) |
| Yes | 7 (3.8) |
| Hospitalised for UC, n (%) |  |
| No | 139 (75.1) |
| Yes | 46 (24.9) |
| Prior screening colonoscopy, n (%) |  |
| No | 140 (75.7) |
| Yes | 45 (24.3) |
| Number of previous anti-TNFα agents, n (%) |  |
| 1 | 152 (82.2) |
| 2 | 31 (16.8) |
| 3 | 2 (1.1) |

GLM = golimumab; Q1 = first quartile; Q3 = third quartile; SD = standard deviation; TNFα = tumour necrosis factor α; UC = ulcerative colitis.

Note: Study cohorts were not mutually exclusive. Patients could qualify for >1 cohort if they met all applicable criteria. Cohort entry, including due to switching, corresponded to the date of first recorded use (i.e., baseline, based on the start of the prescribed treatment course) of one of the cohort-defining therapies.

^a^ Maximum extent of disease reflects the value for this patient ascertained on 30 March 2022, when data extraction occurred. This value may not reflect the actual maximal disease extent at the time of cohort entry. In ENEIDA data, there is only 1 value of this variable per patient; this variable is not date stamped and is subject to continual updating to reflect the maximum extent of disease reached.

Table S-14. Colectomy Due to Intractable Disease Subgroup Analyses

| Subgroup and exposure category comparison | Poisson model  IRR (95% CI) | Cox model  HR (95% CI) |
| --- | --- | --- |
| Concomitant TP at baseline |  |  |
| Without concomitant TP (N = 1,724) |  |  |
| GLM only vs. other anti-TNFα agents only | 0.11 (0.01-0.80) | 0.12 (0.02-0.88) |
| (GLM + other anti-TNFα agents) vs. other anti-TNFα agents only | 2.34 (0.32-17.16) | 2.53 (0.34-18.79) |
| GLM only vs. (GLM + other anti-TNFα agents) | 0.05 (0.00-0.76) | 0.05 (0.00-0.77) |
| With concomitant TP (N = 1,277) |  |  |
| GLM only vs. other anti-TNFα agents only | 1.05 (0.30-3.71) | 1.06 (0.30-3.67) |
| (GLM + other anti-TNFα agents) vs. other anti-TNFα agents only | 19.84 (4.54-86.77) | 10.64 (2.36-48.00) |
| GLM only vs. (GLM + other anti-TNFα agents) | 0.05 (0.01-0.32) | 0.10 (0.02-0.61) |
| Prior anti-TNFα agent therapy |  |  |
| No history of prior anti-TNFα agent therapy (N = 572) |  |  |
| GLM only vs. other anti-TNFα agents only | 0.70 (0.13-3.89) | 0.90 (0.16-4.97) |
| (GLM + other anti-TNFα agents) vs. other anti-TNFα agents only | 6.02 (0.67-54.18) | 3.66 (0.41-33.04) |
| GLM only vs. (GLM + other anti-TNFα agents) | 0.12 (0.01-1.29) | 0.25 (0.02-2.78) |
| Experienced users of anti-TNFα agents (N = 2,429) |  |  |
| GLM only vs. other anti-TNFα agents only | 0.26 (0.06-1.10) | 0.23 (0.06-0.96) |
| (GLM + other anti-TNFα agents) vs. other anti-TNFα agents only | 8.07 (1.96-33.31) | 6.23 (1.49-26.07) |
| GLM only vs. (GLM + other anti-TNFα agents) | 0.03 (0.00-0.23) | 0.04 (0.01-0.27) |
| First anti-TNFα agent used in comparator cohort |  |  |
| First therapy in comparator cohort was infliximab^a^ (N = 2,355) |  |  |
| GLM only vs. other anti-TNFα agents only | 0.26 (0.09-0.74) | 0.26 (0.09-0.72) |
| (GLM + other anti-TNFα agents) vs. other anti-TNFα agents only | 4.26 (1.03-17.62) | 3.70 (0.88-15.47) |
| GLM only vs. (GLM + other anti-TNFα agents) | 0.06 (0.01-0.34) | 0.07 (0.01-0.39) |
| First therapy in comparator cohort was adalimumab^a^ (N = 1,789) |  |  |
| GLM only vs. other anti-TNFα agents only | 0.89 (0.26-3.07) | 0.89 (0.26-3.05) |
| (GLM + other anti-TNFα agents) vs. other anti-TNFα agents only | NE | 10.18 (1.24-83.42) |
| GLM only vs. (GLM + other anti-TNFα agents) | 0.06 (0.01-0.54) | 0.09 (0.01-0.79) |

CI = confidence interval; GLM = golimumab; HR = hazard ratio; IRR = incidence rate ratio; NE = not estimable; TNFα = tumour necrosis factor α; TP = thiopurines.

^a^ All GLM-only use was included in this comparison with a subgroup of the comparator.

Table S-15. Summary of Sensitivity Analyses

| Comparison | Poisson model  IRR (95% CI) | Cox model  HR (95% CI) |
| --- | --- | --- |
| Colectomy due to intractable disease: risk window from exposure through end of follow-up | | |
| GLM vs. other anti-TNFα agents | 0.72 (0.39-1.31) | 0.90 (0.81-1.00) |
| Colectomy due to intractable disease, advanced colorectal neoplasia, or death: composite outcome | | |
| GLM vs. other anti-TNFα agents | NP | 0.72 (0.42-1.26) |
| GLM vs. TP | NP | 1.78 (0.93-3.41) |

CI = confidence interval; GLM = golimumab; HR = hazard ratio; IRR = incidence rate ratio; NP = not performed; TNFα = tumour necrosis factor α; TP = thiopurine; UC = ulcerative colitis.

Note: the analysis was adjusted for age group, UC duration in years, prior treatment with cyclosporine, and sex.

Figure S-1. Cumulative Incidence of Outcomes by Exposure Category

**
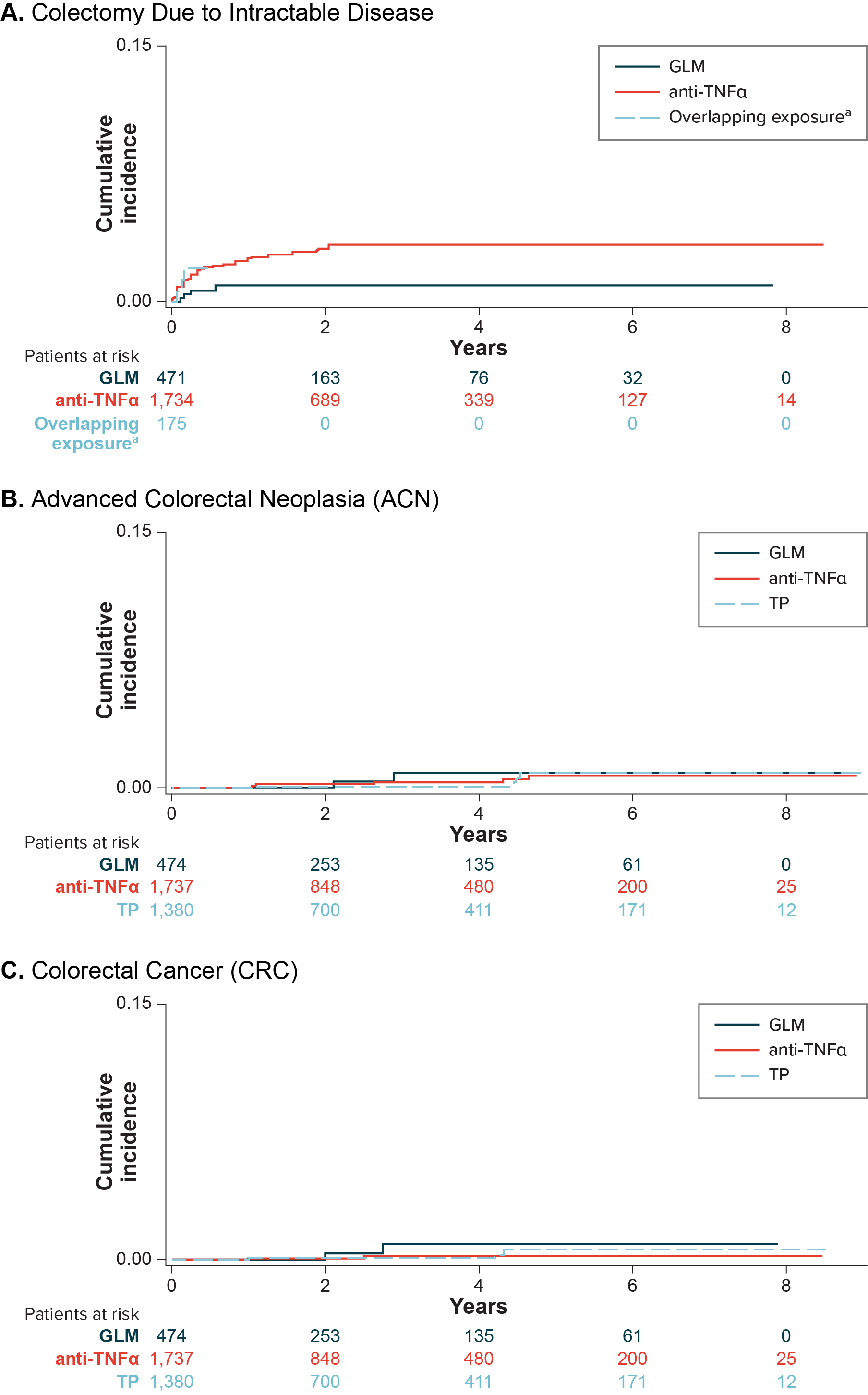
**

anti-TNFɑ = other anti–tumour necrosis factor alpha agents; GLM = golimumab; TP = thiopurine.

Notes: Colectomies due to intractable disease were identified during the period up to 90 days after each therapy discontinuation.

^a^ Overlapping exposure to both GLM and anti-TNFα agents.

# Supplementary References

1. Magro F, Gionchetti P, Eliakim R, et al. Third European evidence-based consensus on diagnosis and management of ulcerative colitis. part 1: definitions, diagnosis, extra-intestinal manifestations, pregnancy, cancer surveillance, surgery, and ileo-anal pouch disorders. *J Crohns Colitis.* 2017;11(6):649-670. doi:<http://dx.doi.org/10.1093/ecco-jcc/jjx008>.

2. Van Assche G, Dignass A, Bokemeyer B, et al. Second European evidence-based consensus on the diagnosis and management of ulcerative colitis part 3: special situations. *J Crohns Colitis.* 2013;7(1):1-33. doi:<http://dx.doi.org/10.1016/j.crohns.2012.09.005>.

3. Bursac Z, Gauss CH, Williams DK, Hosmer DW. Purposeful selection of variables in logistic regression. *Source Code Biol Med.* 2008;3:17. doi:<http://dx.doi.org/10.1186/1751-0473-3-17>.

4. Spinelli A, Bonovas S, Burisch J, et al. ECCO guidelines on therapeutics in ulcerative colitis: surgical treatment. *J Crohns Colitis.* 2021;16(2):179-189. doi:<http://dx.doi.org/10.1093/ecco-jcc/jjab177>.
